# Doctors

**Published:** 1858-05

**Authors:** 


### DOCTORS.

Among the greatest and most real benefactors of the race, next to a faithful clergyman, is an educated physician; for it is to him the world is indebted for the alleviation and removal of calamities which well may make angels weep. It is to the medical teacher the world of humanity owes it, that the insane, the idiotic, and the fatuitous, are treated with sympathetic tenderness, and consideration, instead of stripes, and bludgeons, and chains! It is through the labors of medical men, that the blind, and deaf, and dumb are half relieved of their infirmities; and a still further step has been taken in the *Pennsylvania Farming School for Feeble-minded Children*, according to that excellent paper, which we only sometimes see, *The Philadelphia North American*, by which it appears that children, who have hitherto been left to helpless and disgusting idiocy, their weakness increasing with their years, are, under the operation of this system of training, made at least like human beings. Children who were almost repulsive become, by a judicious mode of teaching, attentive to their own persons, observe a proper manner of eating, and cultivate the decencies and even courtesies of life; while the exhibitions of affection which they show to those who have them in charge are among the most beautiful and touching evidences of the power of love and kindness to humanize and elevate: showing that mind always exists, although clouded, repressed, or entirely overlaid by bodily defects; and that for the amelioration of the physical ills of humanity, that man or woman lives to purpose who bequeaths to such an object a liberal portion of their worldly substance; but nobler still are they, and wiser, who are their own executors, and live to see the results of their beneficence, and thus provoke themselves to greater charities. There is no idiot child, however apparently hopeless a case, for which something may not be done. The managers of this Institution are selected from every religious society, the household being under the direction of Dr. Isaac N. Kenlin, and Dr. Joseph Parish. Surely there is not a nobler institution on earth.

